# Penta­lanthanum zinc diplumbide, La_5_Zn_1−*x*_Pb_2+*x*_ (*x* ≃ 0.6)

**DOI:** 10.1107/S1600536813033618

**Published:** 2013-12-18

**Authors:** Igor Oshchapovsky, Volodymyr Pavlyuk, Grygoriy Dmytriv, Bernd Harbrecht

**Affiliations:** a Department of Inorganic Chemistry, Ivan Franko Lviv National University, Kyryla i Mefodia str. 6, 79005 Lviv, Ukraine; b Department of Chemistry and Materials Science Centre, Philipps University of Marburg, Hans-Meerwein-Strasse 4, 35032 Marburg, Germany

## Abstract

The title non-stoichiometric penta­lanthanum zinc diplumbide, La_5_Zn_1−*x*_Pb_2+*x*_ (*x* ≃ 0.6), was prepared from the elements in an evacuated silica ampoule. It adopts the Nb_5_Sn_2_Si-type structure (space group *I*4/*mcm*, Pearson symbol *tI*32), a ternary ordered superstructure of the W_5_Si_3_ type. Among the four independent crystallographic positions, three are fully occupied by La (Wyckoff 16*k*), La (4*b*), and Pb (8*h*) and one is occupied by a statistical mixture [occupancy ratio 0.394 (12):0.606 (12)] of Zn and Pb (4*a*). The structure is constructed by face-sharing 10-vertex polyhedra around the unmixed Pb sites. These fragments enclose channels of *trans*-face-sharing tetra­gonal anti­prisms occupied by the disordered Zn and Pb sites.

## Related literature   

For general background to electronic structure calculations, see: Andersen *et al.* (1986 ▶); Becke & Edgecombe (1990 ▶); Dronskowski & Blöchl (1993 ▶); Lange (1999 ▶); Nowak *et al.* (1991 ▶). For related structures, see: Aronsson (1955 ▶); Oshchapovsky *et al.* (2011*a* ▶,*b* ▶, 2012*a* ▶,*b* ▶); Stetskiv *et al.* (2012 ▶). For isotypic structures, see: Horyn & Lukaszewich (1970 ▶).

## Experimental   

### 

#### Crystal data  

La_5_Zn_0.394_Pb_2.606_
*M* *_r_* = 1259.40
Tetragonal, 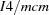

*a* = 12.7630 (18) Å
*c* = 6.3680 (13) Å
*V* = 1037.3 (3) Å^3^
*Z* = 4
Mo *K*α radiation
μ = 63.04 mm^−1^
*T* = 153 K
0.04 × 0.02 × 0.01 mm

#### Data collection  

Bruker P4 CCD diffractometer
Absorption correction: multi-scan (*XSCANS*; Bruker, 1999) *T*_min_ = 0.332, *T*_max_ = 0.525
3933 measured reflections
365 independent reflections
352 reflections with *I* > 2σ(*I*)
*R*_int_ = 0.122

#### Refinement  

*R*[*F* ^2^ > 2σ(*F* ^2^)] = 0.024
*wR*(*F* ^2^) = 0.058
*S* = 1.25
352 reflections
18 parameters
Δρ_max_ = 2.55 e Å^−3^
Δρ_min_ = −1.27 e Å^−3^

### 

Data collection: *XSCANS* (Bruker, 1999 ▶); cell refinement: *XSCANS*; data reduction: *XSCANS*; program(s) used to solve structure: *SHELXS97* (Sheldrick, 2008 ▶); program(s) used to refine structure: *SHELXL97* (Sheldrick, 2008 ▶); molecular graphics: *DIAMOND* (Brandenburg, 2006 ▶) and *VESTA* (Momma & Izumi, 2008 ▶); software used to prepare material for publication: *publCIF* (Westrip, 2010 ▶).

## Supplementary Material

Crystal structure: contains datablock(s) I, New_Global_Publ_Block. DOI: 10.1107/S1600536813033618/hb7160sup1.cif

Structure factors: contains datablock(s) I. DOI: 10.1107/S1600536813033618/hb7160Isup2.hkl

Additional supporting information:  crystallographic information; 3D view; checkCIF report

## Figures and Tables

**Table 1:** Bond lengths, negative iCOHP^*a*^ values and distance contractions in the La_5_Zn_1−*x*_Pb_2+*x*_ compound

Bond	Length (δ in Å)	-iCOHP^*a*^ (eV)	Contraction^*b*^ (%) 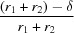 100%
La1—La1	3.611	0.59	3.4
La1—La1	3.825	0.45	−2.3
La1—La1	3.997	0.40	−6.9
La1—La2	4.088	0.40	−9.3
La1—La1	4.193	0.32	−12.1
La2I—La2	3.184	1.26	14.9
			
La1—Zn4	3.365	0.26	−5.2
			
La2—Pb3	3.319	1.10	7.0
La1—Pb3	3.339	1.09	6.5
La1—Pb3	3.484	0.93	2.4
La1—Pb3	3.681	0.72	−3.1
			
Zn4—Zn4	3.184	−0.49	−19.7

Notes: (*a*) integrated Crystal Orbital Hamiltonian Population (see Dronskowski & Blöchl, 1993 ▶); calculated negative iCOHP values enable qualitative estimation of energies of two-center bonds; (*b*) based on metallic radii of elements *R*(La) = 1.86 Å *R*(Zn) = 1.33 Å and *R*(Pb) = 1.70 Å.

**Fig. 1.**
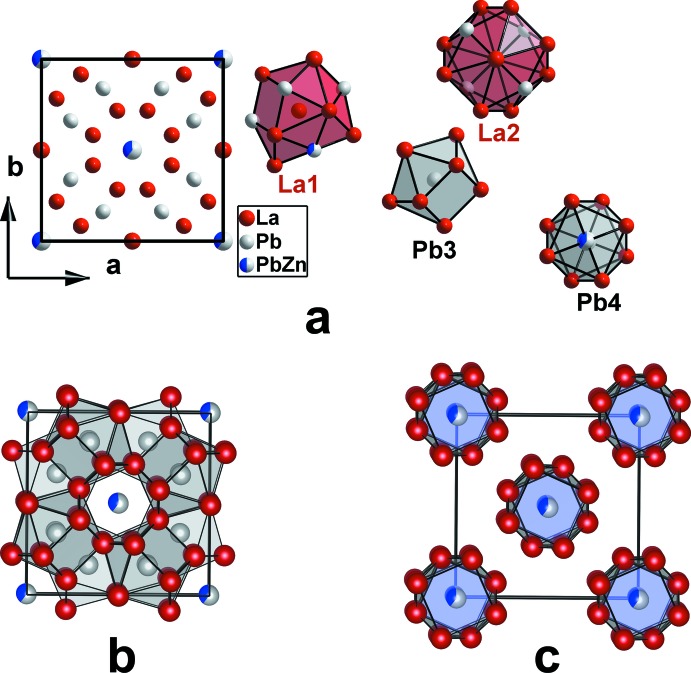
Details of the crystal structure of La5Zn1 - xPb2 + x: Projection of the unit cell and coordination polyhedra (a); building blocks (b); channels in the structure (c).

**Fig. 2.**
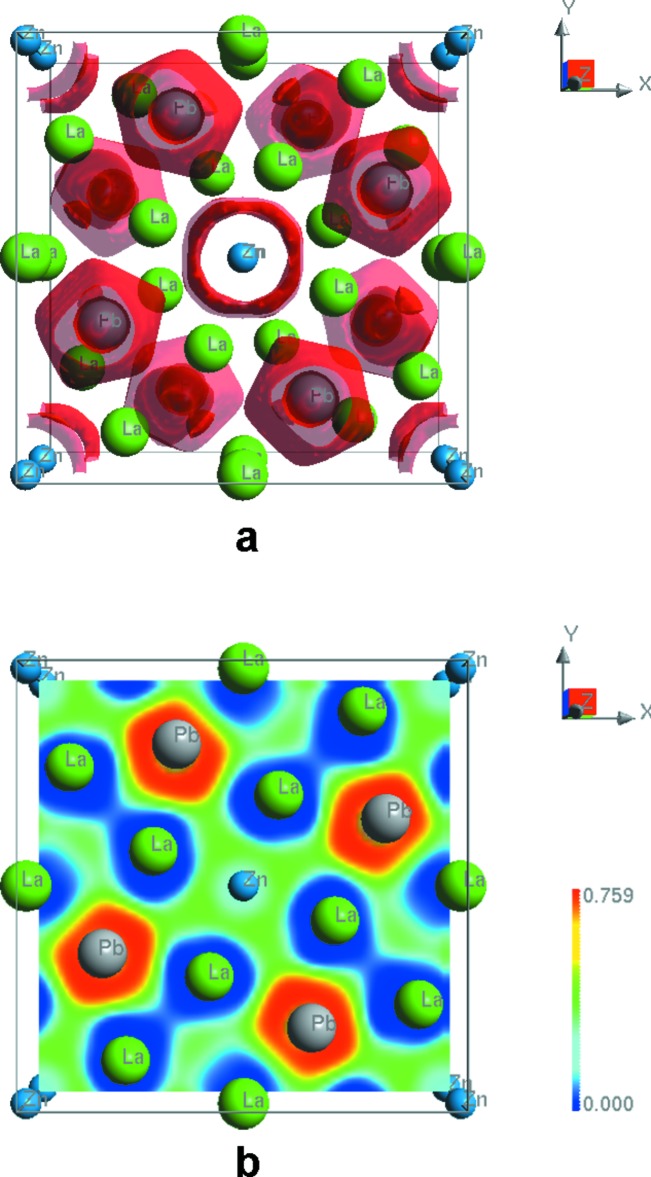
Results of ELF calculations for the La5Zn1 - xPb2 + xcompound: Isosurfaces drawn at 0.45 level (a); section [001] drawn at z=1/2 (b).

**Fig. 3.**
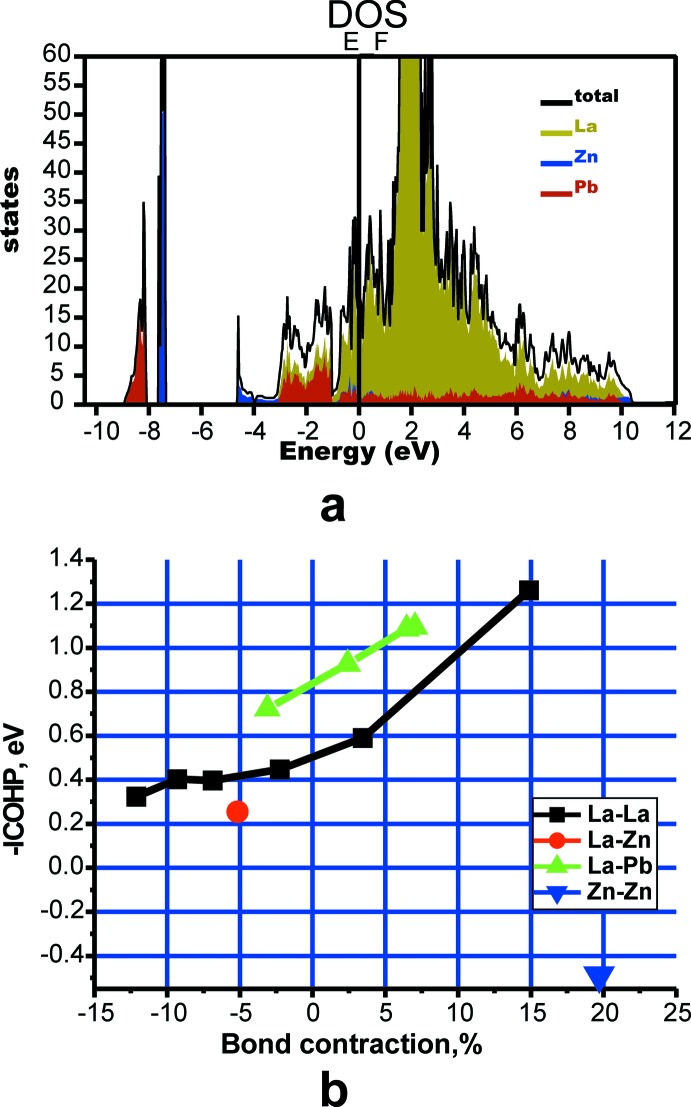
Density of states plot (a); Comparison of bond contractions and -iCOHP values in the title compound (b).

## supplementary crystallographic information

### 1. Comment

The title compound, (I), adopts a Nb_5_Sn_2_Si (Horyn & Lukaszewich, 1970) type structure (space group I4/*mcm*, Pearson symbol tI32), a ternary ordered superstructure of the W_5_Si_3_ type (Aronsson, 1955). A relevant detail of the structure of (I) is presented in Fig. 1.

The coordination polyhedron of the La1 atoms is a 15-vertices polyhedron (CN=15). The nearest neighbours of La2 atoms form 14-vertices polyhedra with CN=14 (see Fig. 1a). The 10-vertices polyhedra around Pb3 can be seen as the major building block of the structure. The face-sharing 10-vertices polyhedra form a three-dimensional framework encasing channels of face-sharing tetragonal antiprisms which in turn accommodate Zn4 and Pb4 with C·N. 10 in a statistical way (see Fig. 1 b, 1c).

This work continues the investigation of {La,Tb}-Zn-{Sn,Pb} ternary systems. In previous articles the bonding interactions in the compounds LaZn_4_ (Oshchapovsky *et al.*, 2012*a*), LaZn_5_ (Oshchapovsky *et al.*, 2012*b*), LaZn_12.37_ (Oshchapovsky *et al.*, 2011*b*) and TbLi_1 -_*_x_*Zn*_x_*Sn_2_ (Stetskiv *et al.*, 2012) were estimated on the basis of electronic structure calculations using the TB-LMTO-ASA program package (Andersen *et al.*, 1986; Nowak *et al.*, 1991) with the additional implementation of two algorithms, the electron localization function (ELF) (Becke & Edgecombe, 1990) and – for quantifying nearest-neighbour bonding interactions – crystal orbital Hamilton population (COHP) (Dronskowski & Blöchl, 1993) analyses. Results arising from these analyses were put in relation to selected crystallographic data of La_5_Zn_2_Sn (Oshchapovsky *et al.*, 2011*a*), namely a differential electronic density map obtained from diffraction data.

In all above mentioned compounds bonding interactions evolve similarly. Atoms of the rare-earth elements usually form metallic bonds with a certain ionic component as they donate part of their electrons to other atoms. The atoms of d- and *p*-metals, namely zinc and tin, accept these electrons used for covalent bonds among each other. This outcome can be easily explained by the higher electronegativity of zinc and tin compared with rare-earth and alkaline metals (Lange, 1999). Judged on the basis of integrated COHP, (iCOHP) values, *R*-{Zn,Sn} bonds are significantly weaker than {Zn,Sn}-{Zn,Sn} bonds. Latter mostly show significant bond length contraction compared to the next contacts in the elemental metals. The differential electronic density map of the structure of La_5_Zn_2_Sn, obtained from experimental diffraction data, indicate donation of electrons to zinc and tin atoms in the similar way as in other compounds, for which electronic structure calculations were performed.

The results of electronic structure calculations for (I) are given in Fig. 2 and 3. As an algorithm for calculating electronic structures for substances with mixed-occupied sites is not implemented in TB-LMTO-ASA package, a fictitious, crystallographically ordered phase was introduced by assuming the statistically occupied Zn/Pb site solely occupied by Zn without change of other crystallographic data.

Similar to R—Zn binary and R—Zn—Sn ternary compounds, large values of the electron localization function (ELF) around Pb3 and Zn4 (originally mixed-occupied) are found. In accordance with similar results for previously cited, structurally related compounds, these findings are interpreted as resulting from donation of electrons from lanthanum atoms to zinc and lead atoms (see Fig. 2a,b).

The values of ELF around Pb3 atoms are significantly larger than around Zn4 atoms, which indicate that bonds between Pb3 and their neighbours should be significantly stronger than bonds between Zn4 atoms and their neighbours. The calculated iCOHP values enable qualitative estimation of energies of two-center bonds and confirm previous assumption. Negative values of iCOHP together with bond lengths and bond contractions are given in Table.

The DOS plot indicates metallic conductivity for the ordered model compound (see Fig. 3a) with small dip at -1 eV relative to Fermi level. Based on a rigid band approximation it can be suggested that these features don't change qualitatively for the title compound. A small dip in the DOS plot near Fermi level could be indirect proof of donation of electrons from lanthanum atoms towards zinc and lead atoms.

The graph negative iCOHP values *versus*. distance contraction shown in Fig. 3 b substantiates the assumption that the polyhedra around Pb3 are indeed the building blocks of the structure if seen as condensed into the given three-dimensional framework by sharing faces. Apparently, this interpretation is not only useful from a crystallographic point of view but also supported by the hierarchy of bonding interactions in the solid: La—Pb bonds are stronger and show larger distance contractions than most La—La bonds. An exception is the particularly strong La2^IX^—La2*^X^* bond enhancing the stability of the framework by connecting adjacent building blocks.

### 2. Experimental

A small irregular grey single-crystal of the title compound of suitable quality was isolated from an alloy with composition La_7_ZnPb_2_ prepared in the course of a systematic investigation of the ternary La—Zn—Pb system. The preparation process was according to that described for La_5_Zn_2_Sn (Oshchapovsky *et al.*, 2011*a*).

The sample was prepared by melting pieces of pure metals (99.9% La, 99.999% Zn, 99.99% Pb) in an evacuated silica ampoule in a resistance furnace with subsequent annealing at 600°C for 30 days. Final phase analysis revealed the presence of the title compound in samples with composition La_7_ZnPb_2_ together with other lanthanum-rich ternary alloys. The latter samples, however, had not reached completely the liquid state, since the use of silica ampoules constraints the maximum temperature to 900°C. This limitation may hamper complete equilibration of the sample.

### 3. Refinement

The fractional site occupancies of Pb4 and Zn4 were constrained to sum to unity.

### Crystal data

**Table:**

La_5_Pb_2.606_Zn_0.394_	*D*_x_ = 8.068 Mg m^−^^3^
*M**_r_* = 1259.40	Mo *K*α radiation, λ = 0.71073 Å
Tetragonal, *I*4/*m**c**m*	Cell parameters from 3933 reflections
Hall symbol: -I 4 2c	θ = 4.5–27.9°
*a* = 12.7630 (18) Å	µ = 63.04 mm^−^^1^
*c* = 6.3680 (13) Å	*T* = 153 K
*V* = 1037.3 (3) Å^3^	Irregularly shaped, metallic grey
*Z* = 4	0.04 × 0.02 × 0.01 mm
*F*(000) = 2041.6	

### Data collection

**Table:**

Bruker P4 CCD diffractometer	365 independent reflections
Radiation source: fine-focus sealed tube	352 reflections with *I* > 2σ(*I*)
Graphite monochromator	*R*_int_ = 0.122
ω scans	θ_max_ = 27.9°, θ_min_ = 4.5°
Absorption correction: multi-scan (*XSCANS*; Bruker, 1999)	*h* = −16→16
*T*_min_ = 0.332, *T*_max_ = 0.525	*k* = −15→16
3933 measured reflections	*l* = −8→8

### Refinement

**Table:**

Refinement on *F*^2^	Primary atom site location: structure-invariant direct methods
Least-squares matrix: full	Secondary atom site location: difference Fourier map
*R*[*F*^2^ > 2σ(*F*^2^)] = 0.024	*w* = 1/[σ^2^(*F*_o_^2^) + 23.7905*P*] where *P* = (*F*_o_^2^ + 2*F*_c_^2^)/3
*wR*(*F*^2^) = 0.058	(Δ/σ)_max_ = 0.012
*S* = 1.25	Δρ_max_ = 2.55 e Å^−^^3^
352 reflections	Δρ_min_ = −1.27 e Å^−^^3^
18 parameters	Extinction correction: *SHELXL97* (Sheldrick, 2008), Fc^*^=kFc[1+0.001xFc^2^λ^3^/sin(2θ)]^-1/4^
0 restraints	Extinction coefficient: 3948 (389)

### Special details

**Table:**

Geometry. All e.s.d.'s (except the e.s.d. in the dihedral angle between two l.s. planes) are estimated using the full covariance matrix. The cell e.s.d.'s are taken into account individually in the estimation of e.s.d.'s in distances, angles and torsion angles; correlations between e.s.d.'s in cell parameters are only used when they are defined by crystal symmetry. An approximate (isotropic) treatment of cell e.s.d.'s is used for estimating e.s.d.'s involving l.s. planes.
Refinement. Refinement of *F*^2^ against ALL reflections. The weighted *R*-factor *wR* and goodness of fit *S* are based on *F*^2^, conventional *R*-factors *R* are based on *F*, with *F* set to zero for negative *F*^2^. The threshold expression of *F*^2^ > σ(*F*^2^) is used only for calculating *R*-factors(gt) *etc*. and is not relevant to the choice of reflections for refinement. *R*-factors based on *F*^2^ are statistically about twice as large as those based on *F*, and *R*- factors based on ALL data will be even larger.

### Fractional atomic coordinates and isotropic or equivalent isotropic displacement parameters (Å^2^)

**Table:**

	*x*	*y*	*z*	*U*_iso_*/*U*_eq_	Occ. (<1)
La1	0.08312 (9)	0.21698 (9)	0.0000	0.0229 (4)	
La2	0.0000	0.5000	0.2500	0.0188 (6)	
Pb3	0.16124 (5)	0.66124 (5)	0.0000	0.0187 (4)	
Pb4	0.0000	0.0000	0.2500	0.0231 (9)	0.606 (12)
Zn4	0.0000	0.0000	0.2500	0.0231 (9)	0.394 (12)

### Atomic displacement parameters (Å^2^)

**Table:**

	*U*^11^	*U*^22^	*U*^33^	*U*^12^	*U*^13^	*U*^23^
La1	0.0150 (6)	0.0150 (6)	0.0386 (9)	0.0005 (4)	0.000	0.000
La2	0.0171 (6)	0.0171 (6)	0.0222 (12)	0.000	0.000	0.000
Pb3	0.0137 (4)	0.0137 (4)	0.0287 (7)	−0.0013 (3)	0.000	0.000
Pb4	0.0142 (8)	0.0142 (8)	0.0410 (16)	0.000	0.000	0.000
Zn4	0.0142 (8)	0.0142 (8)	0.0410 (16)	0.000	0.000	0.000

### Geometric parameters (Å, º)

**Table:**

La1—Pb3^i^	3.3394 (14)	La2—La1^iii^	4.0875 (12)
La1—Zn4^ii^	3.3658 (11)	Pb3—La2^iii^	3.3173 (9)
La1—Pb4^ii^	3.3658 (11)	Pb3—La1^xiv^	3.3394 (14)
La1—Pb4	3.3658 (11)	Pb3—La1^xv^	3.3394 (14)
La1—Pb3^iii^	3.4846 (12)	Pb3—La1^iii^	3.4846 (12)
La1—La1^iv^	3.608 (2)	Pb3—La1^iv^	3.4846 (12)
La1—Pb3^v^	3.6807 (8)	Pb3—La1^xii^	3.6807 (8)
La1—Pb3^vi^	3.6807 (8)	Pb3—La1^xvi^	3.6807 (8)
La1—La1^vii^	3.8261 (13)	Pb3—La1^xi^	3.6807 (8)
La1—La1^viii^	3.8261 (13)	Pb3—La1^xvii^	3.6807 (8)
La1—La1^ix^	3.9969 (14)	Pb4—Zn4^xviii^	3.1840 (7)
La2—La2^x^	3.1840 (7)	Pb4—Zn4^ii^	3.1840 (6)
La2—La2^iii^	3.1840 (6)	Pb4—Pb4^xviii^	3.1840 (7)
La2—Pb3^iii^	3.3173 (9)	Pb4—Pb4^ii^	3.1840 (6)
La2—Pb3	3.3173 (9)	Pb4—La1^xix^	3.3658 (11)
La2—Pb3^v^	3.3173 (9)	Pb4—La1^ii^	3.3658 (11)
La2—Pb3^xi^	3.3173 (9)	Pb4—La1^viii^	3.3658 (11)
La2—La1^xii^	4.0875 (12)	Pb4—La1^xx^	3.3658 (11)
La2—La1^xi^	4.0875 (12)	Pb4—La1^xxi^	3.3658 (11)
La2—La1^xiii^	4.0875 (12)	Pb4—La1^xxii^	3.3658 (11)
La2—La1^v^	4.0875 (12)	Pb4—La1^xxiii^	3.3658 (11)
			
Pb3^i^—La1—Zn4^ii^	97.62 (3)	La1^xii^—La2—La1	149.92 (3)
Pb3^i^—La1—Pb4^ii^	97.62 (3)	La1^xi^—La2—La1	98.725 (6)
Zn4^ii^—La1—Pb4^ii^	0.0	La1^xiii^—La2—La1	107.88 (3)
Pb3^i^—La1—Pb4	97.62 (3)	La1^v^—La2—La1	98.725 (6)
Zn4^ii^—La1—Pb4	56.46 (2)	La1^iii^—La2—La1	134.156 (16)
Pb4^ii^—La1—Pb4	56.46 (2)	La2^iii^—Pb3—La2	57.36 (2)
Pb3^i^—La1—Pb3^iii^	165.81 (4)	La2^iii^—Pb3—La1^xiv^	137.584 (15)
Zn4^ii^—La1—Pb3^iii^	94.87 (3)	La2—Pb3—La1^xiv^	137.584 (15)
Pb4^ii^—La1—Pb3^iii^	94.87 (3)	La2^iii^—Pb3—La1^xv^	137.584 (15)
Pb4—La1—Pb3^iii^	94.87 (3)	La2—Pb3—La1^xv^	137.584 (15)
Pb3^i^—La1—La1^iv^	57.30 (2)	La1^xiv^—Pb3—La1^xv^	65.40 (4)
Zn4^ii^—La1—La1^iv^	143.576 (15)	La2^iii^—Pb3—La1^iii^	73.83 (2)
Pb4^ii^—La1—La1^iv^	143.576 (15)	La2—Pb3—La1^iii^	73.83 (2)
Pb4—La1—La1^iv^	143.576 (15)	La1^xiv^—Pb3—La1^iii^	75.81 (4)
Pb3^iii^—La1—La1^iv^	108.51 (2)	La1^xv^—Pb3—La1^iii^	141.21 (2)
Pb3^i^—La1—Pb3^v^	79.94 (3)	La2^iii^—Pb3—La1^iv^	73.83 (2)
Zn4^ii^—La1—Pb3^v^	147.35 (3)	La2—Pb3—La1^iv^	73.83 (2)
Pb4^ii^—La1—Pb3^v^	147.35 (3)	La1^xiv^—Pb3—La1^iv^	141.21 (2)
Pb4—La1—Pb3^v^	91.356 (15)	La1^xv^—Pb3—La1^iv^	75.81 (4)
Pb3^iii^—La1—Pb3^v^	93.10 (3)	La1^iii^—Pb3—La1^iv^	142.98 (5)
La1^iv^—La1—Pb3^v^	60.650 (17)	La2^iii^—Pb3—La1^xii^	120.60 (3)
Pb3^i^—La1—Pb3^vi^	79.94 (3)	La2—Pb3—La1^xii^	71.26 (2)
Zn4^ii^—La1—Pb3^vi^	91.356 (15)	La1^xiv^—Pb3—La1^xii^	69.21 (3)
Pb4^ii^—La1—Pb3^vi^	91.356 (15)	La1^xv^—Pb3—La1^xii^	100.06 (3)
Pb4—La1—Pb3^vi^	147.35 (3)	La1^iii^—Pb3—La1^xii^	64.48 (2)
Pb3^iii^—La1—Pb3^vi^	93.10 (3)	La1^iv^—Pb3—La1^xii^	119.919 (19)
La1^iv^—La1—Pb3^vi^	60.650 (17)	La2^iii^—Pb3—La1^xvi^	71.26 (2)
Pb3^v^—La1—Pb3^vi^	119.78 (3)	La2—Pb3—La1^xvi^	120.60 (3)
Pb3^i^—La1—La1^vii^	122.81 (3)	La1^xiv^—Pb3—La1^xvi^	69.21 (3)
Zn4^ii^—La1—La1^vii^	55.363 (14)	La1^xv^—Pb3—La1^xvi^	100.06 (3)
Pb4^ii^—La1—La1^vii^	55.363 (14)	La1^iii^—Pb3—La1^xvi^	64.48 (2)
Pb4—La1—La1^vii^	102.64 (3)	La1^iv^—Pb3—La1^xvi^	119.919 (19)
Pb3^iii^—La1—La1^vii^	60.24 (3)	La1^xii^—Pb3—La1^xvi^	119.78 (3)
La1^iv^—La1—La1^vii^	113.086 (18)	La2^iii^—Pb3—La1^xi^	120.60 (3)
Pb3^v^—La1—La1^vii^	150.47 (3)	La2—Pb3—La1^xi^	71.26 (2)
Pb3^vi^—La1—La1^vii^	55.274 (16)	La1^xiv^—Pb3—La1^xi^	100.06 (3)
Pb3^i^—La1—La1^viii^	122.81 (3)	La1^xv^—Pb3—La1^xi^	69.21 (3)
Zn4^ii^—La1—La1^viii^	102.64 (3)	La1^iii^—Pb3—La1^xi^	119.919 (19)
Pb4^ii^—La1—La1^viii^	102.64 (3)	La1^iv^—Pb3—La1^xi^	64.48 (2)
Pb4—La1—La1^viii^	55.363 (14)	La1^xii^—Pb3—La1^xi^	58.70 (3)
Pb3^iii^—La1—La1^viii^	60.24 (3)	La1^xvi^—Pb3—La1^xi^	167.71 (4)
La1^iv^—La1—La1^viii^	113.086 (18)	La2^iii^—Pb3—La1^xvii^	71.26 (2)
Pb3^v^—La1—La1^viii^	55.274 (16)	La2—Pb3—La1^xvii^	120.60 (3)
Pb3^vi^—La1—La1^viii^	150.47 (3)	La1^xiv^—Pb3—La1^xvii^	100.06 (3)
La1^vii^—La1—La1^viii^	112.64 (6)	La1^xv^—Pb3—La1^xvii^	69.21 (3)
Pb3^i^—La1—La1^ix^	59.42 (3)	La1^iii^—Pb3—La1^xvii^	119.919 (19)
Zn4^ii^—La1—La1^ix^	53.576 (15)	La1^iv^—Pb3—La1^xvii^	64.48 (2)
Pb4^ii^—La1—La1^ix^	53.576 (15)	La1^xii^—Pb3—La1^xvii^	167.71 (4)
Pb4—La1—La1^ix^	99.20 (3)	La1^xvi^—Pb3—La1^xvii^	58.70 (3)
Pb3^iii^—La1—La1^ix^	124.98 (2)	La1^xi^—Pb3—La1^xvii^	119.78 (3)
La1^iv^—La1—La1^ix^	90.0	Zn4^xviii^—Pb4—Zn4^ii^	180.0
Pb3^v^—La1—La1^ix^	138.92 (3)	Zn4^xviii^—Pb4—Pb4^xviii^	0.0
Pb3^vi^—La1—La1^ix^	51.362 (18)	Zn4^ii^—Pb4—Pb4^xviii^	180.0
La1^vii^—La1—La1^ix^	64.79 (2)	Zn4^xviii^—Pb4—Pb4^ii^	180.0
La1^viii^—La1—La1^ix^	154.150 (9)	Zn4^ii^—Pb4—Pb4^ii^	0.0
La2^x^—La2—La2^iii^	180.0	Pb4^xviii^—Pb4—Pb4^ii^	180.0
La2^x^—La2—Pb3^iii^	118.679 (10)	Zn4^xviii^—Pb4—La1^xix^	61.771 (11)
La2^iii^—La2—Pb3^iii^	61.321 (10)	Zn4^ii^—Pb4—La1^xix^	118.229 (11)
La2^x^—La2—Pb3	118.679 (10)	Pb4^xviii^—Pb4—La1^xix^	61.771 (11)
La2^iii^—La2—Pb3	61.321 (10)	Pb4^ii^—Pb4—La1^xix^	118.229 (11)
Pb3^iii^—La2—Pb3	122.64 (2)	Zn4^xviii^—Pb4—La1^ii^	118.229 (11)
La2^x^—La2—Pb3^v^	61.321 (10)	Zn4^ii^—Pb4—La1^ii^	61.771 (11)
La2^iii^—La2—Pb3^v^	118.679 (10)	Pb4^xviii^—Pb4—La1^ii^	118.229 (11)
Pb3^iii^—La2—Pb3^v^	103.315 (9)	Pb4^ii^—Pb4—La1^ii^	61.771 (11)
Pb3—La2—Pb3^v^	103.315 (9)	La1^xix^—Pb4—La1^ii^	137.93 (4)
La2^x^—La2—Pb3^xi^	61.321 (10)	Zn4^xviii^—Pb4—La1^viii^	61.771 (11)
La2^iii^—La2—Pb3^xi^	118.679 (10)	Zn4^ii^—Pb4—La1^viii^	118.229 (11)
Pb3^iii^—La2—Pb3^xi^	103.315 (9)	Pb4^xviii^—Pb4—La1^viii^	61.771 (11)
Pb3—La2—Pb3^xi^	103.315 (9)	Pb4^ii^—Pb4—La1^viii^	118.229 (11)
Pb3^v^—La2—Pb3^xi^	122.64 (2)	La1^xix^—Pb4—La1^viii^	77.072 (9)
La2^x^—La2—La1^xii^	67.078 (8)	La1^ii^—Pb4—La1^viii^	143.26 (4)
La2^iii^—La2—La1^xii^	112.922 (8)	Zn4^xviii^—Pb4—La1	118.229 (11)
Pb3^iii^—La2—La1^xii^	153.655 (15)	Zn4^ii^—Pb4—La1	61.771 (11)
Pb3—La2—La1^xii^	58.513 (15)	Pb4^xviii^—Pb4—La1	118.229 (11)
Pb3^v^—La2—La1^xii^	101.555 (15)	Pb4^ii^—Pb4—La1	61.771 (11)
Pb3^xi^—La2—La1^xii^	54.961 (15)	La1^xix^—Pb4—La1	72.85 (3)
La2^x^—La2—La1^xi^	67.078 (8)	La1^ii^—Pb4—La1	123.54 (2)
La2^iii^—La2—La1^xi^	112.922 (8)	La1^viii^—Pb4—La1	69.27 (3)
Pb3^iii^—La2—La1^xi^	153.655 (15)	Zn4^xviii^—Pb4—La1^xx^	118.229 (11)
Pb3—La2—La1^xi^	58.513 (15)	Zn4^ii^—Pb4—La1^xx^	61.771 (11)
Pb3^v^—La2—La1^xi^	54.961 (15)	Pb4^xviii^—Pb4—La1^xx^	118.229 (11)
Pb3^xi^—La2—La1^xi^	101.555 (15)	Pb4^ii^—Pb4—La1^xx^	61.771 (11)
La1^xii^—La2—La1^xi^	52.38 (3)	La1^xix^—Pb4—La1^xx^	69.27 (3)
La2^x^—La2—La1^xiii^	112.922 (8)	La1^ii^—Pb4—La1^xx^	77.072 (9)
La2^iii^—La2—La1^xiii^	67.078 (8)	La1^viii^—Pb4—La1^xx^	137.93 (4)
Pb3^iii^—La2—La1^xiii^	54.961 (15)	La1—Pb4—La1^xx^	77.072 (9)
Pb3—La2—La1^xiii^	101.555 (15)	Zn4^xviii^—Pb4—La1^xxi^	118.229 (11)
Pb3^v^—La2—La1^xiii^	153.655 (15)	Zn4^ii^—Pb4—La1^xxi^	61.771 (11)
Pb3^xi^—La2—La1^xiii^	58.513 (15)	Pb4^xviii^—Pb4—La1^xxi^	118.229 (11)
La1^xii^—La2—La1^xiii^	98.725 (6)	Pb4^ii^—Pb4—La1^xxi^	61.771 (11)
La1^xi^—La2—La1^xiii^	149.92 (3)	La1^xix^—Pb4—La1^xxi^	143.26 (4)
La2^x^—La2—La1^v^	67.078 (8)	La1^ii^—Pb4—La1^xxi^	77.072 (9)
La2^iii^—La2—La1^v^	112.922 (8)	La1^viii^—Pb4—La1^xxi^	72.85 (3)
Pb3^iii^—La2—La1^v^	58.513 (15)	La1—Pb4—La1^xxi^	77.072 (10)
Pb3—La2—La1^v^	153.655 (15)	La1^xx^—Pb4—La1^xxi^	123.54 (2)
Pb3^v^—La2—La1^v^	101.555 (15)	Zn4^xviii^—Pb4—La1^xxii^	61.771 (11)
Pb3^xi^—La2—La1^v^	54.961 (15)	Zn4^ii^—Pb4—La1^xxii^	118.229 (11)
La1^xii^—La2—La1^v^	107.88 (3)	Pb4^xviii^—Pb4—La1^xxii^	61.771 (11)
La1^xi^—La2—La1^v^	134.156 (16)	Pb4^ii^—Pb4—La1^xxii^	118.229 (11)
La1^xiii^—La2—La1^v^	55.81 (2)	La1^xix^—Pb4—La1^xxii^	123.54 (2)
La2^x^—La2—La1^iii^	112.922 (8)	La1^ii^—Pb4—La1^xxii^	72.85 (3)
La2^iii^—La2—La1^iii^	67.078 (8)	La1^viii^—Pb4—La1^xxii^	77.072 (9)
Pb3^iii^—La2—La1^iii^	101.555 (15)	La1—Pb4—La1^xxii^	137.93 (4)
Pb3—La2—La1^iii^	54.961 (15)	La1^xx^—Pb4—La1^xxii^	143.26 (4)
Pb3^v^—La2—La1^iii^	153.655 (15)	La1^xxi^—Pb4—La1^xxii^	69.27 (3)
Pb3^xi^—La2—La1^iii^	58.513 (15)	Zn4^xviii^—Pb4—La1^xxiii^	61.771 (11)
La1^xii^—La2—La1^iii^	55.81 (2)	Zn4^ii^—Pb4—La1^xxiii^	118.229 (11)
La1^xi^—La2—La1^iii^	98.725 (6)	Pb4^xviii^—Pb4—La1^xxiii^	61.771 (11)
La1^xiii^—La2—La1^iii^	52.38 (3)	Pb4^ii^—Pb4—La1^xxiii^	118.229 (11)
La1^v^—La2—La1^iii^	98.725 (6)	La1^xix^—Pb4—La1^xxiii^	77.072 (9)
La2^x^—La2—La1	112.922 (8)	La1^ii^—Pb4—La1^xxiii^	69.27 (3)
La2^iii^—La2—La1	67.078 (8)	La1^viii^—Pb4—La1^xxiii^	123.54 (2)
Pb3^iii^—La2—La1	54.961 (15)	La1—Pb4—La1^xxiii^	143.26 (4)
Pb3—La2—La1	101.555 (15)	La1^xx^—Pb4—La1^xxiii^	72.85 (3)
Pb3^v^—La2—La1	58.513 (15)	La1^xxi^—Pb4—La1^xxiii^	137.93 (4)
Pb3^xi^—La2—La1	153.655 (15)	La1^xxii^—Pb4—La1^xxiii^	77.072 (9)

Symmetry codes: (i) −*y*+1, *x*, *z*; (ii) −*x*, −*y*, −*z*; (iii) −*x*, −*y*+1, −*z*; (iv) −*y*+1/2, −*x*+1/2, *z*; (v) *y*−1/2, −*x*+1/2, −*z*+1/2; (vi) *y*−1/2, −*x*+1/2, −*z*−1/2; (vii) −*x*, *y*, −*z*−1/2; (viii) −*x*, *y*, −*z*+1/2; (ix) *y*, *x*, −*z*−1/2; (x) −*x*, −*y*+1, −*z*+1; (xi) −*y*+1/2, *x*+1/2, *z*+1/2; (xii) *x*, −*y*+1, *z*+1/2; (xiii) *y*−1/2, *x*+1/2, −*z*; (xiv) *y*, −*x*+1, −*z*; (xv) −*x*+1/2, *y*+1/2, −*z*; (xvi) *x*, −*y*+1, *z*−1/2; (xvii) −*y*+1/2, *x*+1/2, *z*−1/2; (xviii) −*x*, −*y*, −*z*+1; (xix) *y*, *x*, −*z*+1/2; (xx) *y*, −*x*, −*z*; (xxi) −*y*, *x*, *z*; (xxii) −*y*, −*x*, *z*+1/2; (xxiii) *x*, −*y*, *z*+1/2.

### Bond lengths, negative iCOHP^a^ values and distance contractions in the La_5_Zn_1-x_Pb_2+x_ compound

**Table:**

Bond	Length (Å)	-iCOHP^a^ (eV)	Contraction^b^ (%) (r_1_+r_2_) - δ - 100%r_1_+r_2_
La1I—La1VII	3.611	0.59	3.4
La1I—La1V	3.825	0.45	-2.3
La1I—La1VIII	3.997	0.40	-6.9
La1I—La2IX	4.088	0.40	-9.3
La1I—La2IX	4.088	0.40	-9.3
La1I—La1III	4.193	0.32	-12.1
La1I—La1IV	4.193	0.32	-12.1
La2IX—La2X	3.184	1.26	14.9
La2IX—La1II	4.088	0.40	-9.3
La2IX—La1III	4.088	0.40	-9.3
La2IX—La1IV	4.088	0.40	-9.3
La2IX—La1V	4.088	0.40	-9.3
La2IX—La1VI	4.088	0.40	-9.3
La2IX—La1VII	4.088	0.40	-9.3
La2IX—La1VIII	4.088	0.40	-9.3
			
La1I—Zn4XV	3.365	0.26	-5.2
La1I—Zn4XVI	3.365	0.26	-5.2
La1II—Zn4XV	3.365	0.26	-5.2
La1III—Zn4XV	3.365	0.26	-5.2
La1IV—Zn4XV	3.365	0.26	-5.2
La1V—Zn4XV	3.365	0.26	-5.2
La1VI—Zn4XV	3.365	0.26	-5.2
La1VII—Zn4XV	3.365	0.26	-5.2
La1VIII—Zn4XV	3.365	0.26	-5.2
			
La2IX—Pb3XI	3.319	1.10	7.0
La2IX—Pb3XII	3.319	1.10	7.0
La2IX—Pb3XIII	3.319	1.10	7.0
La2IX—Pb3XIV	3.319	1.10	7.0
La2X—Pb3XI	3.319	1.10	7.0
La1III—Pb3XI	3.339	1.09	6.5
La1VI—Pb3XI	3.339	1.09	6.5
La1VIII—Pb3XI	3.484	0.93	2.4
La1IV—Pb3XI	3.681	0.72	-3.1
La1V—Pb3XI	3.681	0.72	-3.1
			
Zn4XV—Zn4XVI	3.184	-0.49	-19.7

(a) integrated Crystal Orbital Hamiltonian Population. See (Dronskowski & Blöchl, 1993). Calculated negative iCOHP values enable qualitative estimation of energies of two-center bonds. (b) is based on metallic radii of elements R(La)=1.86 Å R(Zn)=1.33 Å R(Pb)=1.70 Å.
